# Differential Viral Distribution Patterns in Reproductive Tissues of *Apis mellifera* and *Apis cerana* Drones

**DOI:** 10.3389/fvets.2021.608700

**Published:** 2021-03-24

**Authors:** Patcharin Phokasem, Wang Liuhao, Poonnawat Panjad, Tang Yujie, Jilian Li, Panuwan Chantawannakul

**Affiliations:** ^1^Graduate School, Chiang Mai University, Chiang Mai, Thailand; ^2^Bee Protection Laboratory, Department of Biology, Faculty of Science, Chiang Mai University, Chiang Mai, Thailand; ^3^Key Laboratory of Pollinating Insect Biology of the Ministry of Agriculture, Institute of Apicultural Research, Chinese Academy of Agricultural Science, Beijing, China; ^4^Environmental Science Research Center, Faculty of Science, Chiang Mai University, Chiang Mai, Thailand

**Keywords:** *Apis mellifera*, *A. cerana*, drone reproductive, CSBV, venereal transmission

## Abstract

Honeybee drones are male bees that mate with virgin queens during the mating flight, consequently transferring their genes to offspring. Therefore, the health of drones affects the overall fitness of the offspring and ultimately the survivability of the colony. Honeybee viruses are considered to be a major threat to the health of honeybees. In the present study, we demonstrated the pattern of common honeybee viruses in various tissues of drones in the western honeybee, *Apis mellifera*, and the eastern honeybee, *Apis cerana*. Drones were collected during the mating flight and analyzed using quantitative real-time (qRT-PCR) to detect the presence of seven honeybee viruses. The qRT-PCR result revealed that three honeybee viruses, namely Black Queen Cell Virus (BQCV), Deformed Wing Virus (DWV), and Chinese Sacbrood Virus (CSBV), were detected in the reproductive tissues of *A. mellifera* and *A. cerana* drones. The results from qRT-PCR showed that the Israeli Acute Paralysis Virus (IAPV) was only detected in *A. mellifera* drone body tissues. Moreover, the prevalence of DWV and BQCV in the drones collected from *A. mellifera* colonies was significantly higher than that of *A. cerana*. In addition, virus multiple infections were higher in *A. mellifera* drones compared to those in *A. cerana*. CSBV was found predominantly in the reproductive tissues of *A. cerana* drones. This study is the first report describing the presence of the CSBV in reproductive tissues of *A. mellifera* drones. Our results may reflect the preference of honeybee viruses in honeybee species and may provide a piece of interesting evidence for understanding the virus transmission in *A. cerana*.

## Introduction

*Apis* species (the western honeybee, *Apis mellifera*, and the eastern honeybee, *Apis cerana*) are economically valuable pollinators of agricultural crops and are also a source of honeybee products such as honey, pollen, royal jelly, wax, venom, and their larvae (1, 2). The recent loss of honeybee colonies and reduced population of native and wild bees as a result of various stressors, including pathogens, pesticides, habitat loss, and lack of nutritional resources, are global concerns (3, 4). In particular, pathogens and parasites that cause infectious diseases have a major impact on the health of honeybees (5). For instance, Deformed Wing Virus (DWV) infection causes wing deformity, decreased body size, and a reduction in life span in adult honeybees (6, 7). To increase the fitness of the colony, newly emerged queens mate with ~15 drones (8, 9). The natural mating process enables viral transmission through semen to the queen and consequently the eggs, resulting in an infection in honeybee colonies and potentially their products (10–14).

More than 20 honeybee viruses have been identified, and studies have characterized the impact on the health of honeybees and on survival of colonies (15). The common honeybee viruses, such as Acute Bee Paralysis Virus (ABPV), Black Queen Cell Virus (BQCV), Chronic Bee Paralysis Virus (CBPV), DWV, Israeli Acute Paralysis Virus (IAPV), Kashmir Bee Virus (KBV), and Sacbrood Virus (SBV), have been found in honeybee colonies (16). However, despite the increasing knowledge of honeybee virus infections, most studies focus on honeybee workers (17–21) and queens (12, 14, 22–24), and there is little evidence of virus infection in the honeybee drones. Previous studies have shown that honeybee viruses are present in drones (11, 24, 25), but little research has focused on a pattern of honeybee virus infection in drones.

This study aimed to provide a better understanding of the virus infection pattern in honeybee drones. Honeybee drones of two honeybee species (*A. mellifera* and *A. cerana*) were collected to (i) characterize the occurrence of major honeybee viruses in drones and (ii) compare the patterns of honeybee virus distribution in the reproductive tissues [mucus glands (secrete the white mucous substance) and seminal vesicles (storage of sperm)] of drones. These studies have provided evidence for understanding insight into virus transmission in *A. mellifera* and *A. cerana* colony.

## Materials and Methods

### Drone Samples

Samples of *A. cerana* drones (*n* = 103) in managed hives were collected from five colonies in Miyun district (Latitude 40.614586, Longitude 117.04722), Beijing, China, and samples of *A. mellifera* drones (*n* = 116) from seven colonies were obtained at the Institute of Apicultural Research (Latitude 39.995372, Longitude 116.211416), Chinese Academy of Agricultural Science (CAAS), Beijing, China in 2016. All *A. cerana* and *A. mellifera* colonies were queenright and kept in standard hives. Drones were collected on the alighting board of a hive during mating flight times of each species (26), following the method reported in an earlier study (27). Ten drones were placed with 20 workers from the same colony in the holding cage (28). All of the holding cages were placed into an incubator at 34°C and 60% RH until dissection.

### Dissection and Sperm Count

After collection, the individual drone was weighed on a digital balance (scale 0.1 mg) for whole-body mass and then immediately dissected for the reproductive organs (seminal vesicles and mucus glands, Supplementary Figure 1) from the abdomen. Drones were pinned on the thorax (dorsal side up) onto the dissection stage, and dissecting scissors were inserted into the sides of the abdomen to cut the reproductive organs. The reproductive organs were placed on the dissection stage, and then the seminal vesicles were cut off the mucus glands with scissors (27, 29). The body, seminal vesicles, and mucus glands were placed into separate 1.5 ml sterile microcentrifuge tubes and stored at −80°C until RNA extraction. For the spermatozoa count, a single seminal vesicle from each drone was transferred into 0.9% NaCl solution. Total spermatozoa from the seminal vesicle were estimated using a hemocytometer (Fuchs-Rosenthal) under a light microscope (29).

### RNA Extraction and cDNA Synthesis

Total RNA was extracted from the remaining body tissues, seminal vesicles, and mucus glands of an individual honeybee drone using TRIzol reagent (Invitrogen, Carlsbad, CA, USA) according to the protocol of the manufacturer and stored in RNase-free water at −80°C. One hundred ng of total RNA was reverse transcribed with random hexamer primers to the first-strand cDNA using a PrimeScript II 1st strand cDNA Synthesis Kit (Takara Bio, Shiga, Japan) as per the instructions of the manufacturer.

### Quantitative Real-Time PCR (qRT-PCR) Amplification for Seven Honeybee Viruses Present in Different Honeybee Drone Tissues

The presence of seven honeybee viruses, namely ABPV, BQCV, CBPV, CSBV, DWV, IAPV, and KBV, was determined by qRT-PCR using the LineGene 9600 series (Bioer Technology, China). Amplification was performed in 20 μl reaction volumes using SYBR Green Master Mix: SYBR *Premix Ex Taq* II (Tli RNase H Plus; Takara Bio, Shiga, Japan), consisting of 10 μl 2x SYBR Premix ExTaq II (Tli RNaseH Plus), 0.8 μl of 10 μM of each primer and β-actin (Supplementary Table 1), 6.4 μl of nuclease-free water, and 2 μl of cDNA. The run cycle was programed to preincubate at 95°C for 3 min to activate DNA Taq polymerase, and then, 40 cycles of denaturation at 95°C for 30 s, annealing at 60°C for 1 min, and extension at 72°C for 30 s, followed by a melt-curve dissociation analysis were performed. The melting curve was examined at the end of the amplification process to ensure the specificity of PCR products. All reactions were performed in triplicate. In addition, the no-template negative control (H_2_O) was run in every qRT-PCR reaction, and the reference gene, β-actin, was used as an internal control. The qRT-PCR data were expressed as the threshold cycle (Cq) value and normalized against the Cq value of the reference gene (β-actin) to the target genes (ΔCq) (30). The end products from qRT-PCR were purified and sequenced to confirm the results. All sequences were compared against sequences published in GenBank and presented in Supplementary Table 2 and Supplementary Figure 2.

### Statistical Analyses

The frequencies of simultaneous infection prevalence of the honeybee virus were compared among different *A. mellifera* and *A. cerana* drone tissues using a Chi-square test. A one-way ANOVA was used to compare relative differences of the honeybee virus across drone tissues from *A. mellifera* and *A. cerana* drones. The significant difference of each honeybee virus between drone tissues was analyzed using a *t*-test. The physical quality of *A. mellifera* and *A. cerana* drone data were shown as means ± SEM. *P*-values of <0.05 were considered significant.

## Results

### Physical Fitness and Sperm Quantity of *A. mellifera* and *A. cerana* Drone Used in This Study

The number of sperms, whole body weight, mucus glands weight, and seminal vesicle weight from *A. mellifera* and *A. cerana* drones were quantified. The sperm quantity indicated that drone samples were mature with *A. mellifera* producing ~6.08 ± 2.10 × 10^6^ sperm cell/ml in the seminal vesicle. The whole body, seminal vesicle, and mucus glands weights of *A. mellifera* were 222.500 ± 20.148, 21.000 ± 6.716, and 3.000 ± 1.648 mg, respectively (Supplementary Table 3). The eastern honeybee, *A. cerana*, produced ~0.47 ± 0.20 × 10^6^ sperm cell/ml in the seminal vesicle. The whole body, seminal vesicle, and mucus glands weights of *A. cerana* were 103.563 ± 12.507, 11.968 ± 6.126, and 1.500 ± 0.900 mg, respectively (Supplementary Table 3).

### Presence of Honeybee Viruses in Tissues of Drones

To investigate the presence of honeybee viruses, the body tissue, seminal vesicle, and mucus gland samples were taken from the same drone samples. A total of 69 honeybee drones were screened for the seven honeybee viruses, namely ABPV, BQCV, CBPV, CSBV, DWV, IAPV, and KBV.

**Figure 1:**
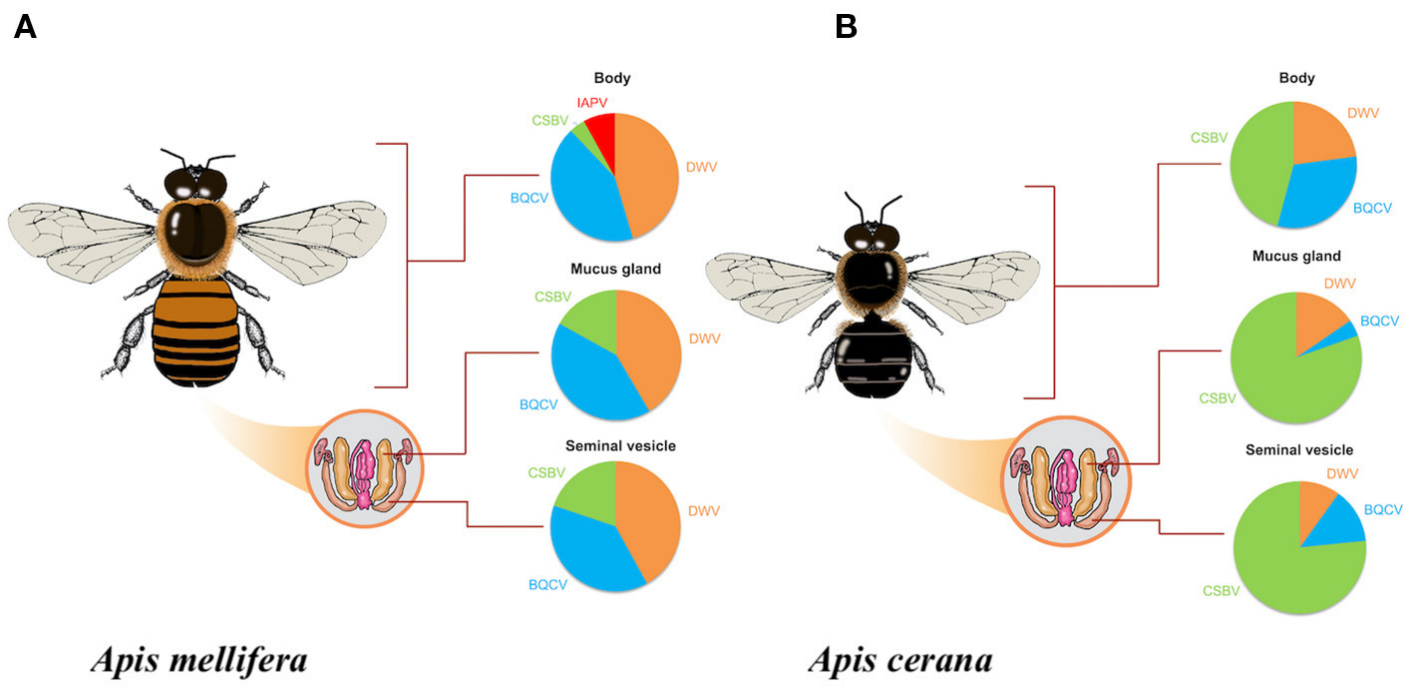
Percentage of honeybee virus infection in each drone tissue **(A)** *Apis mellifera* drones **(B)** *Apis cerana* drones for Black Queen Cell Virus (BQCV); Chinese Sacbrood Virus (CSBV); Deformed Wing Virus (DWV); and Israeli Acute Paralysis Virus (IAPV).

*Apis mellifera* drones: BQCV, CSBV, DWV, and IAPV were detected in one or more *A. mellifera* drone tissues, while ABPV, CBPV, and KBV were not detected (Figure 1A). DWV was found in 79% (*n* = 33/42) of body tissue samples, 71% (*n* = 30/42) of mucus gland samples, and 55% (*n* = 23/42) of seminal vesicle samples. BQCV was found in 74% (*n* = 31/42) of body tissue samples, 71% (*n* = 30/42) of mucus gland samples, and 50% (*n* = 21/42) of seminal vesicle samples. CSBV was found in 7% (*n* = 3/42) of body tissue samples, 29% (*n* = 12/42) of mucus gland samples, and 26% (*n* = 11/42) of seminal vesicle samples. IAPV was found only in 14% (*n* = 6/42) of body tissue samples (Figure 1A).

*Apis cerana* drones: BQCV, CSBV, and DWV were detected in one or more *A. cerana* drone tissues, while ABPV, CBPV, KBV, and IAPV were not detected (Figure 1B). DWV was found in 22% (*n* = 6/27) of body tissue samples, 15% (*n* = 4/27) of mucus gland samples, and 11% (*n* = 3/27) of seminal vesicle samples. BQCV was found in 30% (*n* = 8/27) of body tissue samples, 4% (*n* = 1/27) of mucus gland samples, and 15% (*n* = 4/27) of seminal vesicle samples. CSBV was found in 44% (*n* = 12/27) of body tissue samples, 78% (*n* = 21/27) of mucus gland samples, and 85% (*n* = 23/27) of seminal vesicle samples (Figure 1B).

### The Occurrence of Multiple Honeybee Virus Infections in Tissues of Drones

Simultaneous infections of two or three viruses were detected in *A. mellifera* and *A. cerana* drones. Altogether, the following combination of virus infections was observed: dual infection, BQCV-DWV, BQCV-CSBV, BQCV-IAPV, and DWV-CSBV; triple infection, BQCV-CSBV-DWV. A detailed description of the detection of multiple honeybee virus infections in individual drone samples is summarized in Table 1.

**Table 1:** Frequencies of simultaneous honeybee virus infections in *Apis cerana* and *Apis mellifera* drones.

**No. of viruses**	**Type of infection**	**Drone's tissues**			***P-*value**
		**Body**	**Mucus glands**	**Seminal vesicles**	
***A. cerana***					
0		33% (9/27)	19% (5/27)	11% (3/27)	0.1143
1	DWV	11% (3/27)	4% (1/27)	4% (1/27)	0.4097
	BQCV	7% (2/27)	0% (0/27)	0% (0/27)	0.1221
	CSBV	22% (6/27)	63% (17/27)	67% (18/27)	0.0017
2	DWV+BQCV	4% (1/27)	0% (0/27)	0% (0/27)	0.3589
	DWV+CSBV	4% (1/27)	11% (3/27)	4% (1/27)	0.4097
	BQCV+CSBV	15% (4/27)	4% (1/27)	11% (3/27)	0.3665
3	DWV+BQCV+CSBV	4% (1/27)	0% (0/27)	4% (1/27)	0.5863
***A. mellifera***					
0		5% (2/42)	0% (0/42)	24% (10/42)	0.0003
1	DWV	10% (4/42)	12% (5/42)	17% (7/42)	0.6119
	BQCV	2% (1/42)	19% (8/42)	17% (7/42)	0.0459
	CSBV	0% (0/42)	7% (3/42)	5% (2/42)	0.237
	IAPV	12% (5/42)	0% (0/42)	0% (0/42)	0.005
2	DWV+BQCV	62% (26/42)	40% (17/42)	17% (7/42)	<0.0001
	DWV+CSBV	0% (0/42)	10% (4/42)	5% (2/42)	0.1243
	BQCV+CSBV	0% (0/42)	2% (1/42)	0% (0/42)	0.3709
	BQCV+IAPV	2% (1/42)	0% (0/42)	0% (0/42)	0.1331
3	DWV+BQCV+CSBV	7% (3/42)	10% (4/42)	17% (7/42)	0.3576

*The intensity of red shades showed distinctly different frequencies*.

*Apis mellifera* drones: Honeybee virus infection was detected in 95% (*n* = 40/43) of drone body samples. Among the infected drone body samples, 64% (*n* = 27/42) showed coinfection with two viruses (BQCV-DWV and BQCV-IAPV), and 7% (*n* = 3/42) showed coinfection with three viruses (BQCV-CSBV-DWV). Dual infection occurred in 52% (*n* = 22/42) of drone mucus gland samples. The most prevalent dual infection was the DWV and BQCV pair (40% of samples; *n* = 17/42). Coinfections with the triple infection of BQCV-CSBV-DWV were found in 10% (*n* = 4/42). In seminal vesicles of *A. mellifera* drones, we found higher rates of dual infection (21% of samples; *n* = 9/42) and triple infections (17% of samples; *n* = 7/42). The virus combination results are summarized in Table 1.

*Apis cerana* drones: The most prevalent single virus infection in drone body samples are DWV, BQCV, and CSBV, with frequencies of 11% (*n* = 3/27), 7% (*n* = 2/27), and 22% (*n* = 6/27), respectively. Multiple virus infections were 22% (*n* = 6/27) for dual infection and 4% (*n* = 1/27) for triple infection. Also, the most prevalent coinfection patterns were BQCV-DWV, BQCV-CSBV, and DWV-CSBV, and BQCV-CSBV-DWV. About 70% of the mucus gland contained at least one virus, mainly DWV, and CSBV, whereas two virus infections were found in 15% (*n* = 4/27) of the mucus gland. No viruses were found in 19% (*n* = 5/27). We found that 70% (*n* = 19/27) of seminal vesicle samples showed single infection, the majority of which were CSBV, and the remaining 15% (*n* = 4/27) showed dual-infection (DWV-CSBV, BQCV-CSBV), and 4% (*n* = 1/27) for triple infection (DWV-BQCV-CSBV).

### Levels of Honeybee Viruses in Drone Tissues

**Figure 2:**
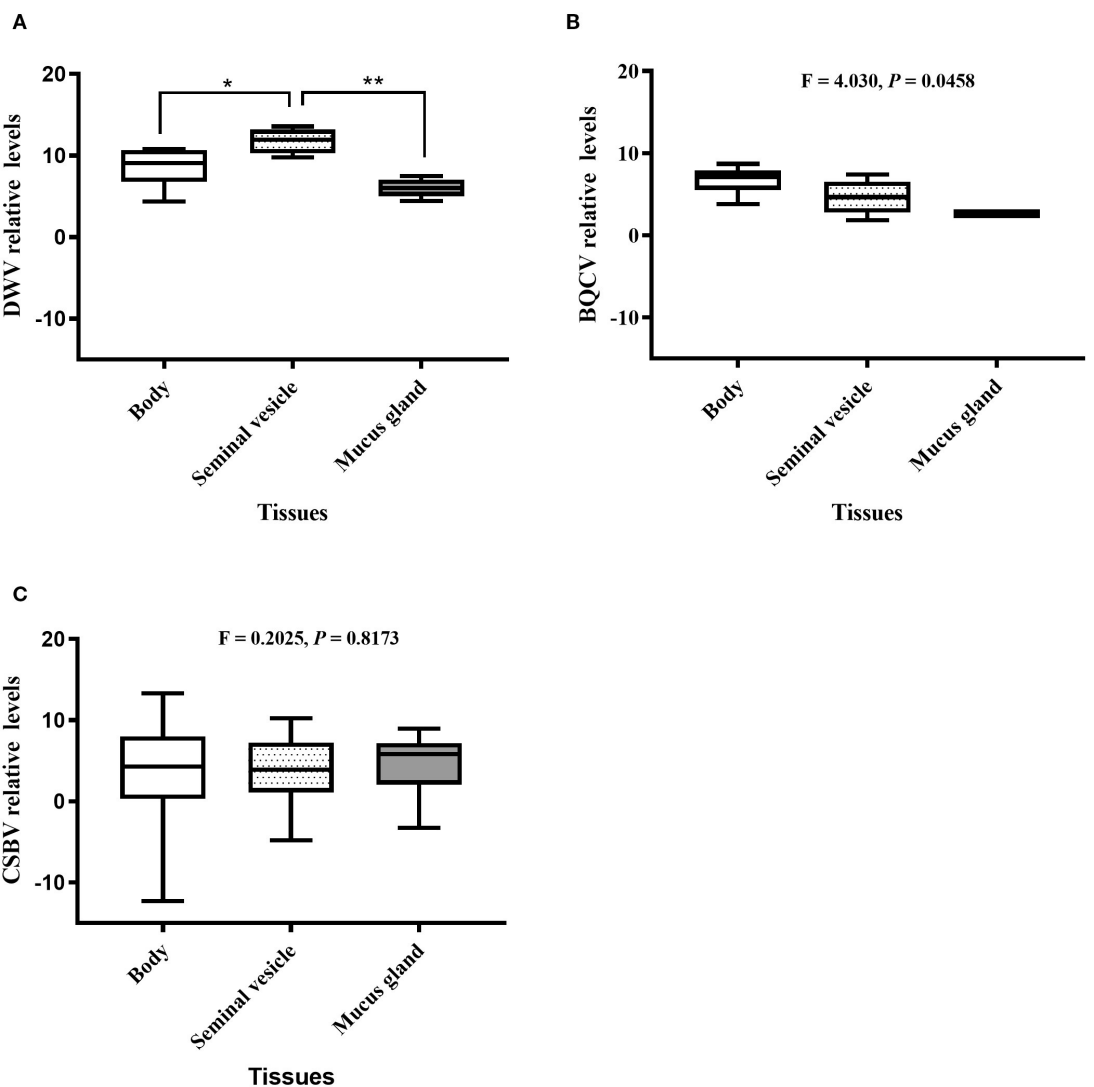
The relative levels of honeybee viruses in each *Apis cerana* drone tissue measured by quantitative real-time (qRT)-PCR. **(A)** DWV relative levels. **(B)** BQCV relative levels. **(C)** CSBV relative levels. *P < 0.05, **P < 0.01.

**Figure 3:**
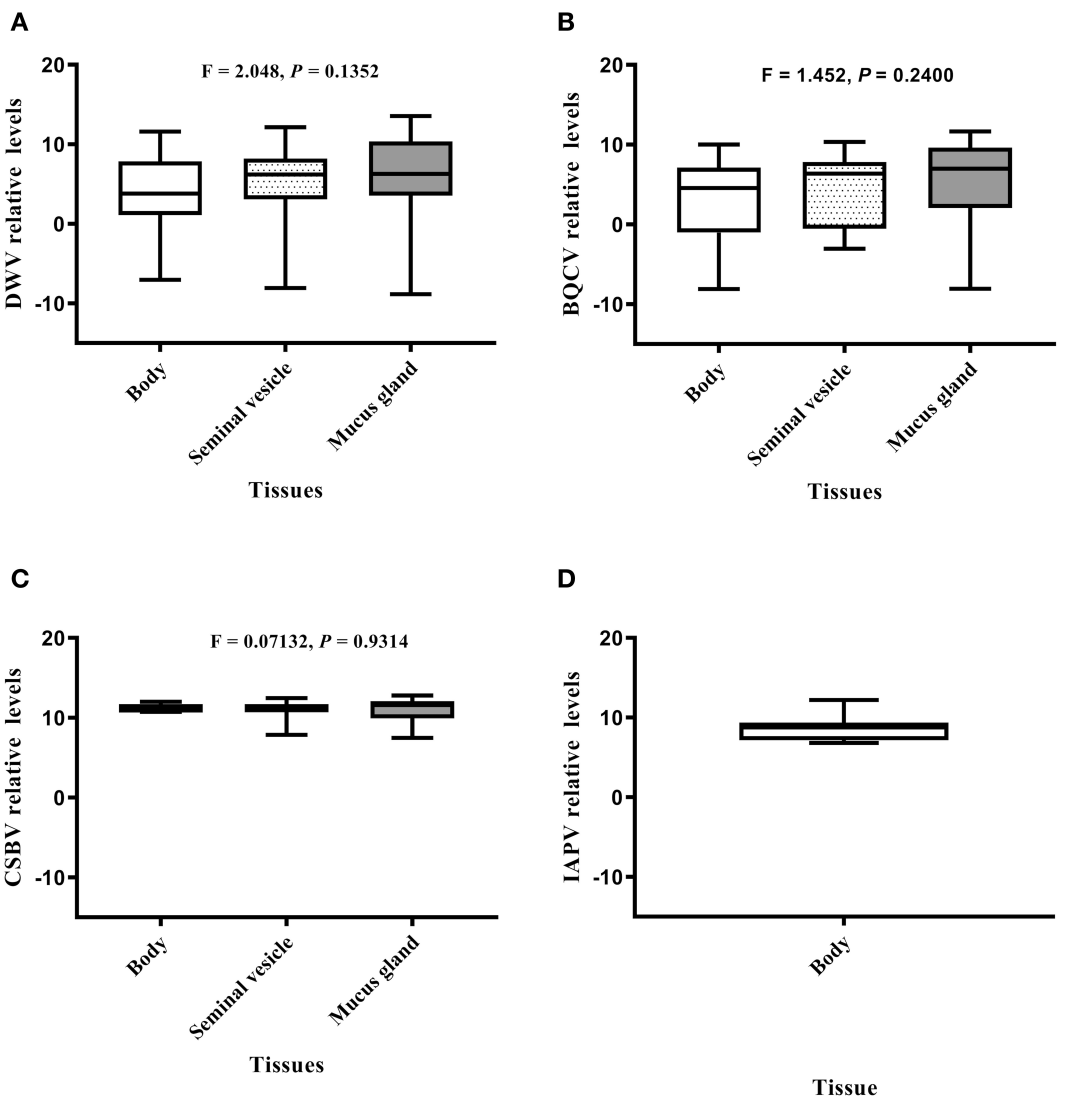
The IAPV relative levels of honeybee viruses in each *Apis mellifera* drone tissue measured by quantitative real-time (qRT)-PCR. **(A)** DWV relative levels. **(B)** BQCV relative levels. **(C)** CSBV relative levels. **(D)** IAPV relative levels.

Relative value viral analyses of *A. mellifera* and *A. cerana* drone tissues showed that all samples were negative for ABPV, CBPV, and KBV. However, four honeybee viruses, BQCV, DWV, CSBV, and IAPV, were detected. In *A. cerana*, DWV levels were significantly different among various tissues (*P* = 0.0016; Figure 2A). Both mucus glands (*P* = 0.0011) and bodies (*P* = 0.0331) had higher DWV levels than seminal vesicles. The levels of BQCV were also significantly different among *A. cerana* drone tissues (*P* = 0.0458; Figure 2B). However, the levels of CSBV displayed no significant difference among *A. cerana* drone tissues (*P* = 0.8173; Figure 2C). In *A. mellifera*, the viral levels (DWV, BQCV, CSBV) showed no significant difference among drone tissues (*P* = 0.1352, 0.2400, 0.9314, respectively; Figure 3).

## Discussion

The present study focused on the viral distribution pattern in drones of two commercial honeybee species. The presence of viruses in *A. cerana* drone tissues was different from those in *A. mellifera* drones. We found that the prevalence of DWV and BQCV in *A. mellifera* drones was relatively high compared to *A. cerana* drones. CSBV was dominant in all tissues of *A. cerana* drones, especially in mucus glands and seminal vesicle tissues. Similarly, BQCV and DWV were the primary viruses detected in *A. mellifera* workers (17), whereas CSBV was the primary virus detected in *A. cerana* workers (18, 31). Previous studies characterized CSBV to exhibit host-specificity and regional diversity (32). Based on our findings, these differences may suggest virus-host specificity and/or tissue types of the drone.

Deformed Wing Virus is known to be the most widespread and is present in all castes of *A. mellifera* (12, 17, 23, 25). Additionally, DWV infections of *A. cerana* workers have been reported in Chinese apiaries (17). Previous studies reported the presence of DWV in seminal vesicles (24), mucus gland (33), and semen (11) of *A. mellifera* drones, suggesting that queens may be infected during natural mating or instrumental insemination (22, 25, 34). If the honeybee queens were infected with DWV, the virus could also be vertically transmitted (35). In addition, the presence of BQCV has recently been found in *A. mellifera* semen obtained from healthy drones (10). Similar to the results of this study, DWV and BQCV were detected in the reproductive organs of drones of both honeybee species, indicating potential sexual transmission of both viruses in honeybees. Lines of evidence have shown that DWV and BQCV infection weakened the colony and could result in colony loss (20, 36). Thus, the detection of DWV and BQCV in the reproductive organs of drones is indicative of overall colony health.

The drone reproductive organ of both honeybee species was positive for CSBV, which is a geographic strain of SBV isolated from Chinese honeybee (*A. cerana*). CSBV poses a serious threat to the *A. cerana* beekeeping industry and crop pollination ecosystem services (21). CSBV causes the Chinese sacbrood disease (CSD), and the 1972 outbreak that occurred in the Guangdong province, China destroyed more than 50% of local Chinese *A. cerana* honeybee colonies. CSD reemerged in Liaoning, China, in 2008 spread to nearly 75% of all *A. cerana* colonies, thereby causing the collapse of Chinese *A. cerana* colonies (37). Recently, CSBV was found to be infecting the ovary of the queen and worker and the semen of the drone and was linked to vertical transmission, food-borne transmission, and venereal transmission in *A. cerana* colonies (18). Our finding is consistent with previous studies and indicated that the prevalence of CSBV in reproductive organs of *A. cerana* drones further substantiated vertical and venereal transmissions. Moreover, our finding showed that the CSBV infection could spread in various drone tissues as it was also found in the body, mucus glands, and seminal vesicles of *A. mellifera* drones. The presence of CSBV in seminal vesicles suggested that CSBV may also be able to transmit in *A. mellifera* colonies through semen. However, further studies will need to be performed to elucidate the virulence of CSBV to *A. mellifera*. Moreover, as both *A. mellifera* and *A. cerana* shared the habitat or were even co-cultured in the same apiaries (38), CSBV could cause infection by crossing the species barrier and potentially kill *A. mellfera* larvae.

We detected high rates of multiple infections in the reproductive tissues of *A. mellifera* drones, in contrast to high rates of single infections in *A. cerana* drones. Multiple infections in *A. mellifera* were likely due to the infestation of ectoparasitic mites and honeybee pathogens [e.g., *Nosema ceranae* (38, 39)], which lead to multiple infections of honeybee viruses in the honeybee colonies. Reports have shown that *Varroa* mite was associated with infections of multiple honeybee viruses, such as DWV, KBV, and IAPV (40–42). Although several studies have revealed that honeybee viruses have been detected in semen (10, 11, 18) and the reproductive system of drones (24, 33), their impacts on drone fertility are still unknown. Therefore, further studies are needed to elucidate the negative effects of viruses on drone health and reproductive fitness.

The worlwide rapid loss of honeybee population and the Colony Collapse Disorder has been recognized since 2006. The beekeepers reported the losses of 50–90% of honeybees, and many areas have suffered from such loss including the US, Europe, and many parts of Asia (2–5). To date, the current status of honeybee populations is continuing to decline worldwide. Honeybee viruses are known to be one of the biotic factors causing colony losses. The finding obtained from our study displayed the honeybee virus patterns in different parts of honeybee drones and a possible sexual transmission route for viruses between colonies of two *Apis* species, which are domesticated in China.

## Data Availability Statement

The original contributions presented in the study are included in the article/Supplementary Material, further inquiries can be directed to the corresponding author/s.

## Ethics Statement

Ethical review and approval was not required for the animal study because this study involved the use of invertebrate animal (honeybees) that were collected at a commercial farm in Miyun district, Beijing, China, and an apiary maintained at the Institute of Apicultural Research, Chinese Academy of Agricultural Science, CAAS, Beijing, China, and no ethical approval was needed.

## Author Contributions

PC and PPh developed the study concept, design in collaboration with JL, performed experiments and analysis, and wrote and revised the manuscript. PPh, PC, and WL participated in the experimental design and interpretation of the data. PPh, PPa, and TY performed experiments. All authors read and approved the manuscript.

## Conflict of Interest

The authors declare that the research was conducted in the absence of any commercial or financial relationships that could be construed as a potential conflict of interest.
