# Supplementary material for: Differential Viral Distribution Patterns in Reproductive Tissues of Apis mellifera and Apis cerana Drones
Source: Front Vet Sci. 2021 Mar 24;8:608700. doi: 10.3389/fvets.2021.608700 (PMC8024463; doi:10.3389/fvets.2021.608700)
Supplement: Supplementary file 1 [file Table_1.DOCX]

Supplementary Material

# Supplementary Tables

Table S1 Primers used in this study.

| Primers | Product size (bp) | Primer sequence (5'-3') | References |
| --- | --- | --- | --- |
| CSBV | 257 | CCACCGACTGTTAAGACGAAT | [1] |
|  |  | GCTGACCCAAAATAGGGAGTT |  |
| IAPV | 158 | GGTGCCCTATTTAGGGTGAGGA | [2] |
|  |  | GGGAGTATTGCTTTCTTGTTGTG |  |
| DWV | 194 | CTTACTCTGCCGTCGCCCA | [3] |
|  |  | CCGTTAGGAACTCATTATCGCG |  |
| BQCV | 140 | TTTAGAGCGAATTCGGAAACA | [4] |
|  |  | GGCGTACCGATAAAGATGGA |  |
| KBV | 69 | CGTCGACCTATTGAAAAAGTTAATCA | [5] |
|  |  | TGAGAAGTCCATTGGTCCATTTG |  |
| CBPV | 97 | GATACCGTCGTCACCCTCATG |  |
|  |  | CGGCAACGGATTCATCAAC |  |
| ABPV | 66 | AAATGATACCGGTGGGCAGAT |  |
|  |  | AAGGTCGTATGTCCGTCTTACCA |  |
| β-actin | 129 | TTGTATGCCAACACTGTCCTTT | [6] |

REFERENCES

1. Shan L, Liuhao W, Jun G, Yujie T, Yanping C, Jie W, et al. Chinese sacbrood virus infection in Asian honey bees (*Apis cerana cerana*) and host immune responses to the virus infection. *J. Invertebr. Pathol.* (2017) 150:63-69. doi:10.1016/j.jip.2017.09.006.

2. Sguazza GH, Reynaldi FJ, Galosi CM, Pecoraro MR. Simultaneous detection of bee viruses by multiplex PCR. *J Virol Methods.* (2013) 194:102-106. doi:10.1016/j.jviromet.2013.08.003.

3. Chen Y, Pettis JS, Feldlaufer MF. Detection of multiple viruses in queens of the honey bee *Apis mellifera* L. *J Invertebr Pathol.* (2005) 90:118-121. doi:10.1016/j.jip.2005.08.005.

4. vanEngelsdorp D, Evans JD, Saegerman C, Mullin C, Haubruge E, Nguyen BK, et al. Colony collapse disorder: a descriptive study. *PLoS ONE.* (2009) 4:e6481. doi:10.1371/journal.pone.0006481.

5. Gauthier L, Tentcheva D, Tournaire M, Dainat B, Cousserans F, Colin ME, et al. Viral load estimation in asymptomatic honey bee colonies using the quantitative RT-PCR technique. *Apidologie.*  (2007) 38:426-435. doi:10.1051/apido:2007026.

6. Chaimanee V, Chantawannakul P, Chen Y, Evans JD, Pettis JS. Differential expression of immune genes of adult honey bee (*Apis mellifera*) after inoculated by Nosema ceranae. *J Insect Physiol.* (2012) 58:1090-1095.
