# Supplementary material for: Differential Viral Distribution Patterns in Reproductive Tissues of Apis mellifera and Apis cerana Drones
Source: Front Vet Sci. 2021 Mar 24;8:608700. doi: 10.3389/fvets.2021.608700 (PMC8024463; doi:10.3389/fvets.2021.608700)
Supplement: Supplementary file 2 [file Table_2.DOCX]

Supplementary Material

# Supplementary Tables

Table S2 Virus sequence alignment by Basic Local Alignment Search Tool (BLAST - NCBI), to confirm the qPCR results.

| Sample | Matching | Max Score | Total Score | Query Cover | E value | **Per. Ident** | Accession |
| --- | --- | --- | --- | --- | --- | --- | --- |
| BQCV_101 | Black queen cell virus isolate 32 capsid protein gene, partial cds | 1195 | 1195 | 99% | 0.0 | 99% | KP7300331 |
| BQCV_122 | Black queen cell virus isolate 07 capsid protein gene, partial cds | 1229 | 1229 | 99% | 0.0 | 99% | KP730008.1 |
| CSBV_20 | Sacbrood virus strain CSBV-JL/China/2014, complete genome | 237 | 237 | 96% | 5e-59 | 94% | KU574661.1 |
| CSBV_135,  CSBV_204 | Sacbrood virus strain AmCSBV-SDLY/China/2016 polyprotein mRNA, complete cds | 261 | 261 | 95% | 8e-66 | 96.25% | MG733283.1 |
| DWV_100 | Deformed Wing Virus isolate Chilensis A1, genomic RNA | 636 | 636 | 97% | 1e-178 | 95% | JQ413340.1 |
| IAPV_192 | Israeli acute paralysis virus RdRP genes for RNA dependent RNA polymerase, structural protein, partial cds, isolate: China-5_iapv | 206 | 206 | 93% | 1e-49 | 98% | AB745498.1 |
