# Supplementary material for: Differential Viral Distribution Patterns in Reproductive Tissues of Apis mellifera and Apis cerana Drones
Source: Front Vet Sci. 2021 Mar 24;8:608700. doi: 10.3389/fvets.2021.608700 (PMC8024463; doi:10.3389/fvets.2021.608700)
Supplement: Supplementary file 3 [file Table_3.DOCX]

Supplementary Material

# Supplementary Table

**Table S3** Means of drone weight, seminal vesicle weight, mucus gland weight and number of sperm of *Apis mellifera* and *A. cerana.*

| Honeybee species | Body weight  (mg) | Mucus gland weight (mg)^1^ | Seminal vesicle weight (mg)^2^ | Sperm count  (×10^6^ cell/ ml)^3^ |
| --- | --- | --- | --- | --- |
| *A. mellifera* | 222.500 ± 20.148  (n = 116) | 21.000 ± 6.716  (n = 77) | 3.000 ± 1.648  (n = 172) | 6.08 ± 2.10  (n = 64) |
| *A. cerana* | 103.563 ± 12.507  (n = 103) | 11.968 ± 6.126  (n = 31) | 1.500 ± 0.900  (n = 197) | 0.47 ± 0.20  (n = 45) |

^1^ Weights are from both mucus glands of individual drones

^2^ Weights are from single seminal vesicles of individual drones

^3^ Counts are from single seminal vesicles of individual drones

^*^ Measured parameters are given as mean ± SE
