# Supplementary material for: Differential Viral Distribution Patterns in Reproductive Tissues of Apis mellifera and Apis cerana Drones
Source: Front Vet Sci. 2021 Mar 24;8:608700. doi: 10.3389/fvets.2021.608700 (PMC8024463; doi:10.3389/fvets.2021.608700)
Supplement: Supplementary file 4 [file Data_Sheet_1.PDF]

Supplementary Material

Supplementary Figure

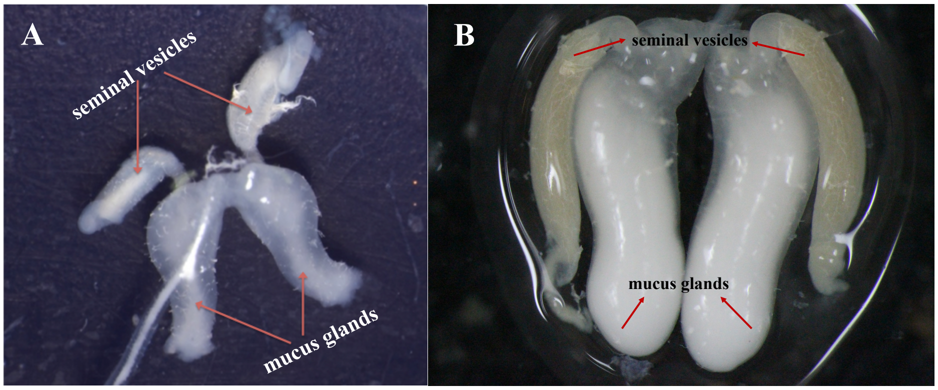

Figure S1 Reproductive organs of honeybee drones (A) *Apis cerana* (B) *Apis mellifera*.
