# Supplementary material for: Differential Viral Distribution Patterns in Reproductive Tissues of Apis mellifera and Apis cerana Drones
Source: Front Vet Sci. 2021 Mar 24;8:608700. doi: 10.3389/fvets.2021.608700 (PMC8024463; doi:10.3389/fvets.2021.608700)
Supplement: Supplementary file 5 [file Data_Sheet_2.PDF]

Supplementary Material

Supplementary Figure

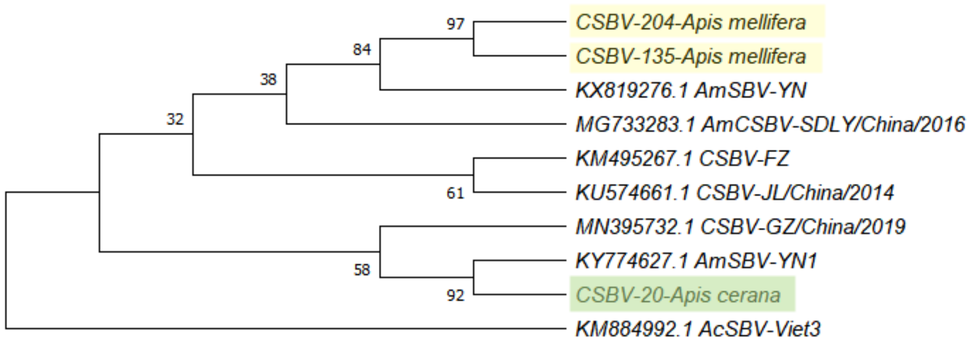

Figure S2 Bootstrap consensus tree of Chinese Sacbrood virus in infected honeybee drones.

Evolutionary analysis by Maximum Likelihood method

The evolutionary history was inferred by using the Maximum Likelihood method and Tamura-Nei model [1]. The bootstrap consensus tree inferred from 1000 replicates [3] is taken to represent the evolutionary history of the taxa analyzed [3]. Branches corresponding to partitions reproduced in less than 50% bootstrap replicates are collapsed. The percentage of replicate trees in which the associated taxa clustered together in the bootstrap test (1000 replicates) are shown next to the branches [3]. Initial tree(s) for the heuristic search were obtained automatically by applying Neighbor-Join and BioNJ algorithms to a matrix of pairwise distances estimated using the Tamura-Nei model, and then selecting the topology with superior log likelihood value. This analysis involved 10 nucleotide sequences. There were a total of 186 positions in the final dataset. Evolutionary analyses were conducted in MEGA X [2]

1. Tamura K. and Nei M. (**1993**). Estimation of the number of nucleotide substitutions in the control region of mitochondrial DNA in humans and chimpanzees. *Molecular Biology and Evolution* **10**:512-526.
2. Kumar S., Stecher G., Li M., Knyaz C., and Tamura K. (**2018**). MEGA X: Molecular Evolutionary Genetics Analysis across computing platforms. *Molecular Biology and Evolution* **35**:1547-1549.
3. Felsenstein J. (**1985**). Confidence limits on phylogenies: An approach using the bootstrap. *Evolution* **39**:783-791.
